# Roles of extracellular vesicles associated non-coding RNAs in Diabetes Mellitus

**DOI:** 10.3389/fendo.2022.1057407

**Published:** 2022-12-22

**Authors:** Benoit R. Gauthier, Nadia Cobo-Vuilleumier, Livia López-Noriega

**Affiliations:** ^1^ Andalusian Center for Molecular Biology and Regenerative Medicine-CABIMER, Junta de Andalucía-University of Pablo de Olavide-University of Seville-Consejo Superior de Investigaciones Científicas (CSIC), Seville, Spain; ^2^ Centro de Investigacion Biomedica en Red de Diabetes y Enfermedades Metabolicas Asociadas (CIBERDEM), Madrid, Spain

**Keywords:** diabetes mellitus, extracellular vesicles, micro-RNAs, long non-coding RNAs, cellular crosstalk

## Abstract

Extracellular vesicles (EVs), especially exosomes (50 to 150 nm), have been shown to play important roles in a wide range of physiological and pathological processes, including metabolic diseases such as Diabetes Mellitus (DM). In the last decade, several studies have demonstrated how EVs are involved in cell-to-cell communication. EVs are enriched in proteins, mRNAs and non-coding RNAs (miRNAs, long non-coding RNAs and circRNAS, among others) which are transferred to recipient cells and may have a profound impact in either their survival or functionality. Several studies have pointed out the contribution of exosomal miRNAs, such as miR-l42-3p and miR-26, in the development of Type 1 and Type 2 DM (T1DM and T2DM), respectively. In addition, some miRNA families such as miR-let7 and miR-29 found in exosomes have been associated with both types of diabetes, suggesting that they share common etiological features. The knowledge about the role of exosomal long non-coding RNAs in this group of diseases is more immature, but the exosomal lncRNA MALAT1 has been found to be elevated in the plasma of individuals with T2DM, while more than 169 lncRNAs were reported to be differentially expressed between healthy donors and people with T1DM. Here, we review the current knowledge about exosomal non-coding RNAs in DM and discuss their potential as novel biomarkers and possible therapeutic targets.

## 1 Introduction

Diabetes Mellitus (DM) is a group of metabolic diseases characterized by chronic hyperglycemia resulting from pancreatic β-cell failure accompanied or not by insulin resistance (1). Type 1 DM (T1DM) stems from the selective destruction of β-cells by the immune system, although many long-term individuals with T1DM still have a residual beta cell mass and changes in β-cell function have also been reported (2, 3). Type 2 DM (T2DM) and gestational DM (GDM) usually appear when β-cells are unable to maintain physiological glucose levels in the presence of insulin resistance (4). Defective insulin secretion -partially caused by a complex crosstalk between different cell-types- is, therefore, a hallmark of most forms of DM (5, 6). These three forms of DM also share an intricate etiology that involves a complex interplay between genetic predisposition and environment (7–9). Hence, identifying factors involved in cell-to-cell communication that may contribute to reduced β-cell mass and function is crucial to understand the pathogenesis of DM and to find a cure.

Extracellular vesicles (EVs) are phospholipid bilayer membrane-enclosed vesicles released from cells into extracellular space and contribute to cell-to-cell communication in a wide range of physiological and pathological processes *in vivo* (10). EVs were initially visualized as “garbage bags’’ released from cells. However, growing evidence indicates that EVs transport cell-derived biological molecules such as nucleic acids (DNA, mRNAs and non-coding RNAs; ncRNAs), proteins, and lipids that may be functionally incorporated by recipient cells. Actually, EVs have been shown to mediate signal exchange between multiple tissues/organs and cell types (11). A pioneer study showed that EVs from rat and human pancreatic islets could contribute to the progression of T1DM since they contained intracellular ß-cell autoantigens (glutamic acid decarboxylase 65, GAD65; insulinoma antigen-2, IA-2; and proinsulin), which were captured by dendritic cells and processed with the subsequent activation of autoreactive T and B cells (12). Besides their roles in mediating intercellular communication, EVs have also been proposed as a source for potential diagnostic and prognostic biomarkers for many diseases since they are easily accessible from different biofluids such as blood (13–16). In this regard, EVs-associated non-coding RNA (ncRNAs) are particularly interesting due to the specificity in their expression pattern. Furthermore, these ncRNAs have been shown to mediate important phenotypic effects in recipient cells, thus they could serve as novel therapeutic targets (17, 18). Actually, the majority of the risk variants for both T1DM and T2DM identified by genome wide studies (GWAS) reside in the non-coding regions of the genome, suggesting that they substantially contribute to the development of diabetes (19). For instance, a major hotspot in GWAS for T2DM and coronary diseases is located in the region of the lncRNA, ANRIL, while polymorphisms in the lncRNA MEG3 alter susceptibility to T1DM in cohorts of European ancestry, where the disease is more prevalent (20–22). Likewise, several polymorphisms in microRNAS such as miR-124 have been associated with T2DM in both Chinese and Italian populations, while let-7 targets are enriched for genes containing SNPs associated with this disease (23, 24). In this review, we summarize the current knowledge about EVs-derived ncRNAs -focusing on micro-RNAs (miRNAs) and long ncRNAs (lncRNAs)- as players in cell-to-cell communication during DM progression. Furthermore, we briefly discuss their potential biomedical applications as biomarkers and therapeutic targets.

## 2 Biogenesis, classification and internalization of EVs

**Figure 1:**
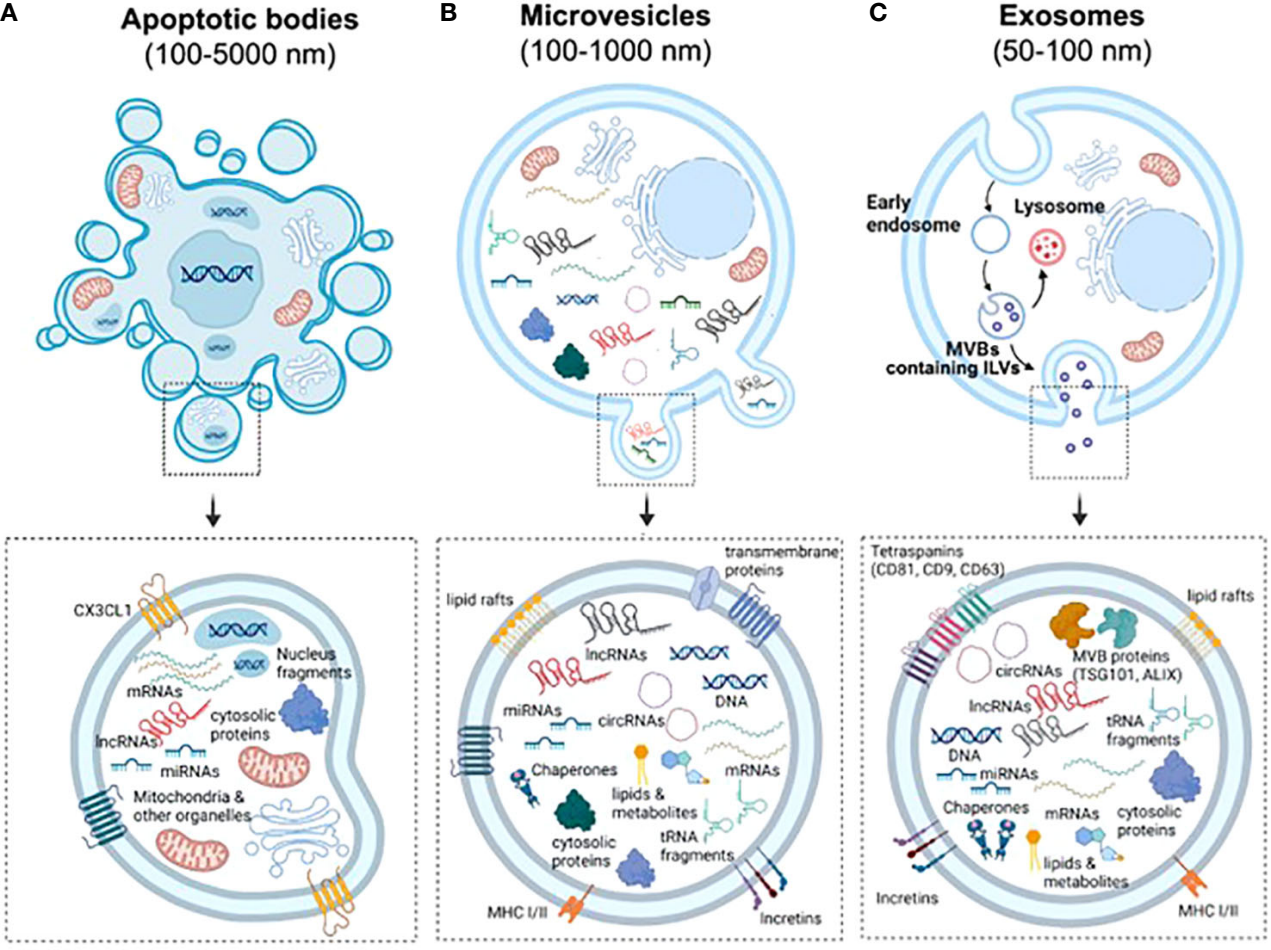
Biogenesis and characteristics of the three main types of EVs. **(A)** Apoptotic bodies are formed by blebbing of the plasma membrane during apoptosis. Similarly to other vesicles, they contain nucleic acids, proteins and different RNA species, but also fragments of the nuclei and full organelles such as mitochondria. **(B)** Microvesicles are formed by budding of the plasma membrane. Therefore, they contain the same transmembrane proteins, lipid rafts and domains than the surface of parental cells. Furthermore, the cargo of EVs include cytosolic proteins, lipids, metabolites, DNA, chaperones, mRNAs and ncRNAs (miRNAs, lncRNAs, cicRNAs and tRNA fragments, among others). **(C)** Exosomes are generated by the endosomal pathway in a process that involves the formation of endocytic vesicles by invagination of the plasma membrane, the inward budding of the endosomal membrane, followed by the formation of intralumenal vesicles and MVBs. Due to their biogenesis, exosomes can be distinguished by an enrichment of tetraspanins and the presence of proteins that form the endosomal sorting complexes required for transport (ESCRTs) required for the transport of MVBs.

EVs are a highly heterogeneous population of naturally occurring membrane- enclosed vesicles which are normally classified into three major categories according to their biogenesis, release mechanism and size: apoptotic bodies (100–5,000 nm), micro-vesicles or ectosomes (100–1,000 nm), and exosomes (50-150 nm) (**Figure 1**) (25). Yet some studies have described other types of EVs such as large oncosomes, which are specifically released by cancer cells and can measure up to10 μm (26). Apoptotic bodies are formed from cellular blebbing and fragmentation during apoptosis (**Figure 1A**). Micro-vesicles arise from the outward budding of the plasma membrane (**Figure 1B**) (27). In contrast, exosomes are formed as part of the endosomal pathway. The process involves the formation of endocytic vesicles by invagination of the plasma membrane, followed by the inward budding of the endosomal membrane, which then produces multivesicular bodies (MVB). MVB can then be targeted to lysosomes, resulting in the degradation of their cargo, or fuse with the plasma membrane, being released to the extracellular matrix as mature exosomes (**Figure 1C**) (28). Distinct EV types display important differences in their cargo and membrane composition. For example, micro-vesicles possess the same extracellular domains of transmembrane proteins than the surface of their parental cell, while exosomes are enriched in tetraspanins and contain several proteins that are specifically required for MVB transport such as the endosomal sorting complex proteins (ESCRT), TSG101 and ALIX (**Figure 1C**). Importantly, differences in EV size and/or membrane composition probably dictate their recognition and internalization by recipient cells (29). Therefore, although we are only starting to appreciate EV diversity, it is vital to fully characterize them, especially if they are meant to be used as mediators of cell communication and delivery tools.

The mechanisms of EV internalization and functional delivery of their cargo into recipient cells are only starting to be understood. EVs have been reported to be internalized by recipient cells through different mechanisms such as clathrin/caveolin-mediated endocytosis, macropinocytosis, lipid-raft-mediated uptake, phagocytosis, membrane fusion and tunneling nanotubes mediating transfer (30, 31). However, different types of EVs may be preferentially internalized by specific mechanisms depending on their characteristics. For example, only exosomes can be internalized by macropinocytosis, as other EVs may be too large to be incorporated through this mechanism (29). In addition, Horibe et al. showed that different cell types may preferentially use different mechanisms to capture exosomes, since two lines of colorectal cancer relied on caveolin-mediated endocytosis, while the lung cancer cell line A549 preferentially incorporated exosomes through macropinocytosis (32).

To what extent EVs uptake is a cell specific process is currently a matter of debate. Indeed, several studies have reported similar efficiencies in the uptake of exosomes between different cancer cell lines and human umbilical vein endothelial cells (HUVECs) (29, 32). In contrast, Zech et al. showed that peritoneal exudate cells incorporated most of the exosomes derived from a metastatic rat pancreatic adenocarcinoma cell line, while thymocytes only internalized a very low proportion of them (33). Similarly, Gomez-Ferrer et al. demonstrated that CD14+ cells were more prone to internalize EVs than other peripheral mononuclear blood cells (34). In an independent study, the authors reported that tetraspanin levels within exosomes influenced whether they were more efficiently incorporated by hematopoietic or endothelial and pancreatic cells (35). These discrepancies probably arise from the heterogeneity in the donor/recipient cells, the specific characteristics of the EVs used and differences in the experimental setup. Nevertheless, these studies highlight the need to further characterize and understand the process of EV biogenesis and uptake before they can be used as therapeutic agents.

Once internalized, EVs can transfer their cargo for either degradation or re-released to the extracellular matrix. It has been proposed that exosomes which fuse with the plasma membrane release their contents into the cytosol, while direct interaction of exosomes with the surface receptors of recipient cells induces downstream signaling cascades. It has been reported that a percentage of the cargo from exosomes is degraded by lysosomes, while another fraction may be re-released to the extracellular matrix, but a significant proportion is functionally transferred to recipient cells (36).

## 3 NcRNAs in EVs

Technical advances in the detection of low-abundance complex RNA in samples have revealed the high diversity of RNA species found in EVs. RNA cargo of EVs is partially representative of parental cells and include mRNAs and ncRNAs such as miRNAs, transfer RNAs (tRNAs) as well as tRNA fragments, Y RNAs, piwi-interacting RNAs (piRNAS), small nucleolar RNAs (snoRNAs), lncRNAs and circular RNA (circRNAs) (37, 38). Nevertheless, there are important differences in the RNA composition/ratio of EVs compared to their parental cells, suggesting that some biotypes are selectively incorporated into EVs (39, 40). Indeed, it seems that EVs are particularly enriched in small ncRNAs (<200 nt), especially miRNAs, although significant levels of lncRNAs (>200 nt) and circRNAs have also been reported. We are only starting to understand how different RNA species are sorted into EVs, but an increasing body of evidence indicate that RNA-biding proteins (RBPs) -such as hnRNPA1, hnRNPA2B1, SYNCRIP or YBX1- are major players in this process (41–43). Villarroya-Beltri et al. showed that the protein HnRNPA2B1 specifically binds to miRNAs packaged in EVs and regulates their loading to exosomes (41). Importantly, a recent study demonstrated that specific short sequences or motifs (EXOmotifs) within RNAs determine whether miRNAs are packaged into EVs or retained within cells. Interestingly, several of these motifs were cell specific, while others were shared among some of the cells tested (hepatocytes, skeletal muscle cells, endothelial cells as well as brown and white adipocytes) (44). Supporting the role of RBPs in the sorting of miRNAs, the motif with stronger enrichment in EVs was shown to interact with two factors, Alyref and Rus, for which repression reduced the presence of the motif-containing miRNAs in EVs (44).

Most studies concerning ncRNAs in EVs have focused in miRNAs, which are short sequences (~20 nucleotides long) that mainly act by inducing target mRNAs degradation or inhibiting protein translation. Numerous studies have reported the functional transfer of miRNAs in EVs between different cell types. In addition, EV-derived miRNA profile in diverse biofluids has been characterized for several human diseases, including T1DM, T2DM and GDM (45–48). Knowledge regarding the function of other small ncRNAs in EVs is more immature since these species have only recently been identified within EVs. For example, tRNA fragments are nearly as abundant as miRNAs in EVs but it is still unclear whether they are functionally transferred to recipient cells (39). In contrast, the packaging of tRNA fragments into EVs may be required for specific cellular processes in parental cells rather than in recipient cells. Indeed, Chiou et al. showed that tRNA fragments suppress T cell activation, thus they are selectively sorted to MBVs for their release during this process (49).

Despite the bias for small ncRNAs, several lncRNAs and circRNAs are enriched in EVs (50, 51). LncRNAs are traditionally defined as transcripts longer than 200 nucleotides with no protein-coding function, although in recent years some lncRNAs have been found to encode micro-peptides (52, 53). The general knowledge of lncRNA regulation and function is still immature compared to other ncRNAs and their high diversity make them very difficult to classify and annotate. The known roles for lncRNAs include modulating promoter-enhancer interactions, chromatin remodeling, modulation of transcription factors’ activity acting as scaffolds or decoys, miRNAs sponging, regulation of alternative splicing and intercellular trafficking (54). CircRNAs are single-stranded RNAs that are covalently linked to form a continuous closed-loop which results from a non-canonical splicing event called backsplicing (55). Similar to lncRNAs, circRNAs regulate the expression of protein coding genes through different mechanisms, although the majority of circRNAs currently described act as miRNA sponges (56). To date, mechanisms regulating lncRNAs and circRNAs sorting into EVs remain largely unknown. LncRNAs harboring motifs that interact with RBPs (ELAVL1 and RBMX) and specific miRNAs (let-7 family members) have been found particularly enriched in prostate cancer-derived EVs. Thus, a model where lncRNAs are sorted in EVs depending on their interaction with RBPs and miRNAS has been proposed (57). Supporting a major role for RBPs in the sorting of lncRNAs into EVs, another study found that exosomal lncRNAs are enriched in GC-rich motifs, which interact with proteins such as PPRC1, RBM4, FUS, and RBM8A, as well as in UAG-containing motifs that bind to HNRNPA1 and HNRNPA2B1 (42, 58, 59). As mentioned previously, two of these RBPs, HnRNPA2B1 and FUS, have also been shown to regulate the sorting of miRNAs into EVs (44) Therefore, it is tempting to speculate that these RNA-binding proteins are master regulators of ncRNAs into EVs. Independently of the mechanism, numerous studies performed in different cell lines indicate that specific lncRNAs and circRNAs are selectively sorted and released in EVs, as the ratio/enrichment of these RNAs significantly differ from parental cells (39, 58, 59). In addition, lncRNAs have been demonstrated to be functionally transferred to recipient cells, where they may regulate different cellular processes such as cellular viability (50, 51). Noteworthy, several lncRNAs and circRNAS have been associated with both T1DM and T2DM and have been shown to play important roles in β-cell function and survival (59–61). Furthermore, several of these lncRNAs, such as MALAT1, GAS5 and TUG1 were also found in exosomes in a number of pathophysiological conditions (62–67).

### 3.1 EV associated ncRNAs involved in autoimmunity during T1D

The pathogenesis of T1DM involves a complex crosstalk between insulin secreting pancreatic β-cells and immune cells (68, 69), which is partially mediated by EVs (70). As such ncRNAs being a constituent of the EVs cargo, could play a key role in the dialogue between pancreatic endocrine cells and the immune system.

**Figure 2:**
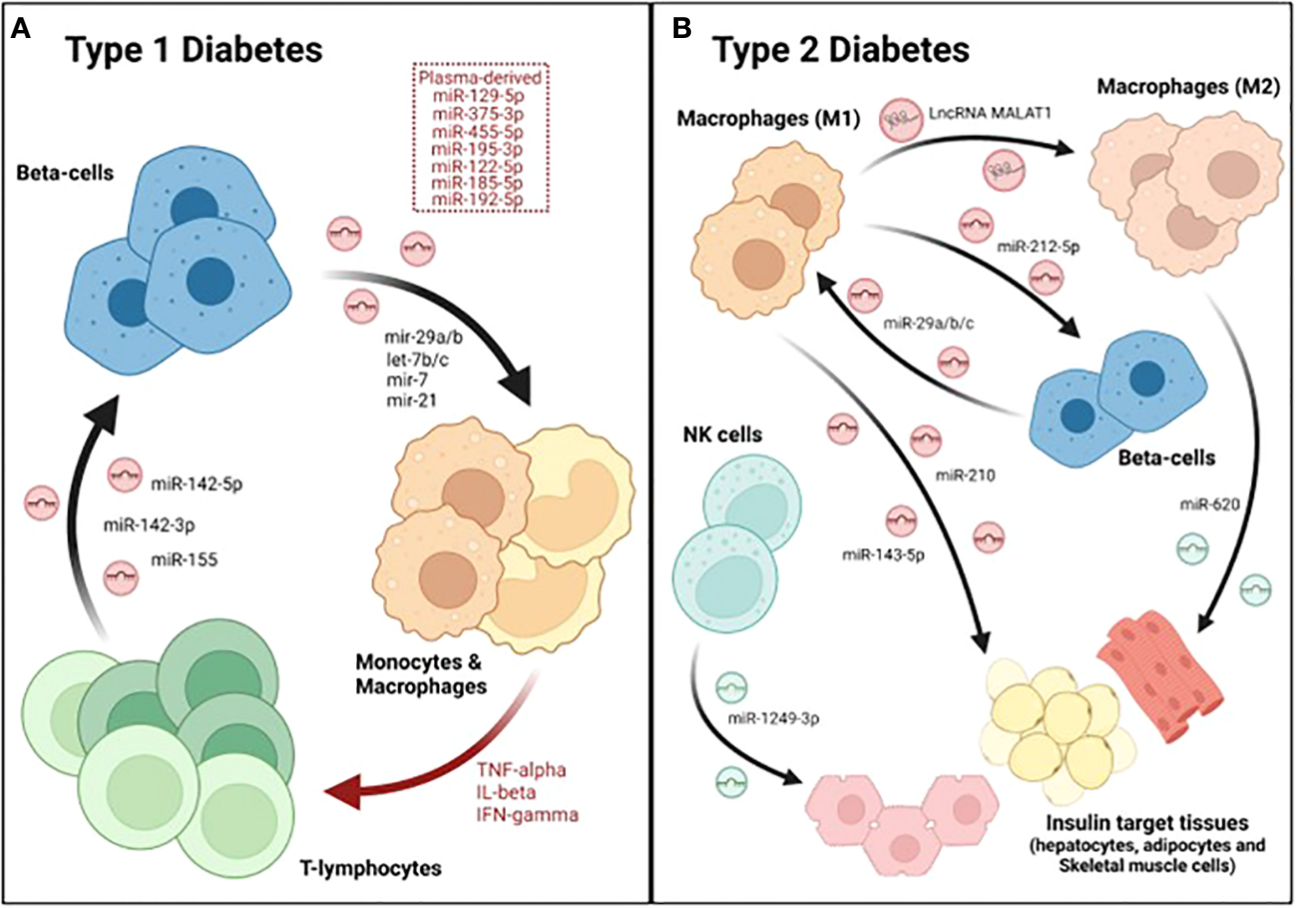
Dialogue between immune and β-cells as well as other metabolic tissues during DM. A crosstalk between immune cells, especially macrophages, pancreatic endocrine cells and insulin-target tissues occurs during the major forms of diabetes. **(A)** During T1DM, β-cells secrete several exosomal miRNAs that stimulate monocytes and macrophages. These antigen presenting cells (APCs) then activate T cells, which also secrete several miRNAs that induce apoptosis in β-cells, initiating a vicious cycle that culminates with the destruction of the majority of β-cell mass. **(B)** In type 2, M1-derived exosomes contain several miRNAs that reduce beta-cell function and/or insulin sensitivity. Furthermore, the lncRNA MALAT1, also found in M1-derived exosomes, polarizes other macrophages towards a M1-like phenotype, further worsening disease progression. In contrast, exosomes released by M2 and NK cells attenuate insulin resistance though the action of miRNAs such as miR-620 and miR-1249-3p, respectively.

In order to determine how inflammation may affect the RNA cargo of human islet-derived EVs, Krishnan et al. performed both total and small RNA sequencing analyses of EVs isolated from human pancreatic islets after cytokine exposure (50 U/ml IL-1β and 1000 U/ml IFN-γ), which was used as a model of T1DM. The authors reported that ~69% of the total long RNA content of EVs derived from both control and cytokine-treated islets was composed by mRNA, while ~21% corresponded to lncRNAs and ~10% mapped to other RNA species. Likewise, the proportion of small RNA species within EVs was not affected by the cytokine treatment. Approximately 20% of the total reads mapped to miRNAs in both experimental groups, while 6.8% corresponded to tRNAs and other species such as piRNAs or snoRNAs represented 0.8% and 0.2%, respectively. Overall, this study reported 31 lncRNAs, 19 miRNAs, 25 piRNAs, 8 snoRNAs, and 20 tRNAs that were differentially expressed in EVs derived from cytokine-treated islets compared to controls (71). Although the function of the different ncRNAs identified was not determined, this data suggested that islet-derived EV associated ncRNAs may contribute to pathogenic mechanisms during the development of T1DM. Supporting this hypothesis, Guay et al. showed that exosomes released from the mouse MIN6B1 β-cell line treated with cytokines (30 ng/ml IFNγ, 10 ng/ml TNFα and 1 ng/ml IL-1β) induced apoptosis in non-stressed β-cells (MIN6B1 and dispersed cells from mouse pancreatic islets). The effect in cell death was blunted after silencing Argonaute 2, a member of the RISC complex required for miRNA action, indicating that the apoptotic signaling was mediated by exosomal miRNAs (72). Another study showed that miR-29b released by MIN6 cell-derived exosomes stimulated TNFα secretion from splenocytes isolated from diabetes-prone NOD mice *in vitro* (**Figure 2A**; **Table 1**) (73). In line with this, Tesovnik and colleagues found several miRNAs, that were upregulated in plasma-derived EVs from T1DM patients (miR-122-5p, miR-192-5p, miR-185-5p, miR-455-5p) and islet-transplanted individuals (miR-375-3p, and miR-129-5p) which induced NK and T-cell early transition proliferation and cytotoxicity in whole blood samples (**Figure 2A**; **Table 1**). These effects were accompanied by an increase in IFN-α IFN-γ, TNF-α, IL-1β, IL-10, IL-6, and MCP-1 levels. However, miRNA mediated effects were blunted when the signaling pathway downstream toll-like-receptor 7/8 (TLR7/8), expressed in monocytes and granulocytes, was deactivated with chloroquine. Therefore, the authors proposed a model in which β-cell-derived EV associated miRNAs are internalized and accumulated in monocytes, where they trigger TLR7/8 mediated response through yet unknown direct targets, leading to the release of specific cytokines and subsequent activation of other subpopulations of the innate and adaptive immune system (76). Nonetheless, a caveat of this study was that EV preparations were not enriched for specific β-cell markers. Therefore, despite their correlation with T1DM and β-cell death/stress after transplantation, it is not possible to confirm whether these miRNAs were specifically upregulated in β-cell derived EVs. Giri and colleages, in contrast, showed the upregulation of other immune stimulatory miRNAs that bind to and directly target TLR (mir-7a, mir-21, mir-29a, let-7b, let7-c) in EVs from MIN6 β-cells when treated with cytokines (**Table 1**) (74). Altogether, this data strongly supports an important role for β-cell derived EV associated miRNAs in the crosstalk between pancreatic endocrine cells and immune cells. In contrast, the role of other ncRNAs, such as lncRNAs that have also been shown to be differentially expressed in β-cell-derived EVs under inflammation, remains to be elucidated.

**Table 1:** EV associated ncRNAs dysregulated in different forms of diabetes.

EV NcRNA	Expression	Disease	Parental cell and disease model	Target/Function	Reference
miR-29	up	T1DM/T2DM	MIN6 treated with cytokines/Mice fed with HFD	Increases TNF alpha secretion in a TLR activation dependent manner/Increases monocyte recruitment and activation through TRAF3	(73–75)
miR-122-5p	up	T1DM	Total blood from subjects with T1DM	Activation of TLR7/8 pathway in monocytes and subsequent NK and T cell proliferation and apoptosis	(76)
miR-192-5p	up	T1DM	Total blood from subjects with T1DM	Activation of TLR7/8 pathway in monocytes and subsequent NK and T cell proliferation and apoptosis	(76)
miR-455-5p	up	T1DM	Total blood from subjects with T1DM	Activation of TLR7/8 pathway in monocytes and subsequent NK and T cell proliferation and apoptosis	(76)
miR-185-5p	up	T1DM	Total blood from subjects with T1DM	Activation of TLR7/8 pathway in monocytes and subsequent NK and T cell proliferation and apoptosis	(76)
mir-7a	up	T1DM	MIN6 treated with cytokines	Bind to TLR7 in innate immune cells	(74)
mir-21	up	T1DM	MIN6 treated with cytokines	Bind to TLR7 in innate immune cells	(74)
let-7b	up/down	T1DM/T2DM	MIN6 treated with cytokines/Serum from subjects with T2DM	Bind to TLR7 in innate immune cells	(74)
let7-c	up	T1DM	MIN6 treated with cytokines	Bind to TLR7 in innate immune cells	(74)
miR-142-3p/-5p	up	T1DM	T lymphocytes NOD mice	Induce Ccl2, Ccl7 and Cxcl10 expression in β-cells through the translocation of NF-κB	(77)
miR-155	up	T1DM	T lymphocytes NOD mice	Induce Ccl2, Ccl7 and Cxcl10 expression in β-cells through the translocation of NF-κB	(77)
miR-16-5p	down	T1DM	Plasma from subjects with T1DM	Inhibits β-cells apoptosis targeting CXCL10	(40, 78)
miRNA-302d-3p	down	T1DM	Plasma from subjects with T1DM	Unknown	(40)
miR-574-5p	down	T1DM/GDM	Plasma from subjects with T1DM and GDM	Targets HDAC9 which promotes adipogenesis	(40, 79, 80)
miR-21-5p	up	T1DM	NOD mice & Serum of T1DM subjects recently diagnosed	Unknown	(81)
IK-208	up	T1DM	Plasma of subjects with T1DM	Unknown	(82)
CANX-210	down	T1DM	Plasma of subjects with T1DM	Unknown	(82)
miR-212-5p	up	T2DM	M1 macrophages from HFD fed mice	Impairs insulin secretion by inhibiting Sirt2	(83)
miR-210	up	T2DM	macrophage RAW264.7 cells incubated with HG concentrations	Inhibition of NDUFA4 and subsequent impaired glucose uptake and mitochondrial function in adipocytes	(84)
miR-143-5p	up	T2DM	M1 macrophages from HFD fed mice	Inhibits Mkp5 expression promoting insulin resistance in hepatocytes	(85)
miR-1249-3p	down	T2DM	NK from lean mice compared to HFD fed mice	Inhibits SKOR1, reducing NF-κB activity and improving insulin resistance in hepatocytes and adipocytes	(86)
MALAT1	up	T2DM	M1 macrophages cultured at HG concentrations	Sponge for miR-150-5p, impairing glucose uptake	(62)
miR-16	up	T2DM	Skeletal-muscle from mice fed with HPD-Evs	Inhibits Ptch1, inducing β-cells proliferation	(87)
mir-222	up	T2DM	Serum and WAT from T2DM human subjects and HFD mice	Inhibits AKT phosphorylation and insulin signaling	(88)
miR-26a	down	T2DM	Serum of overweight subjects and mice fed with HFD	Induces AKT phosphorylation, promoting insulin sensitivity	(62)
lncRNA-p3134	up	T2DM	MIN6 and mouse islets cultured with HG	Activation of the insulin PI3K/Akt/mTOR signaling pathway in β-cells, enhancing GSIS	(89)
miR-326	up	T2DM	Plasma of subjects with T2DM	Predicted to target adiponectin	(90)
let-7a	down	T2DM	Plasma of subjects with T2DM	Unknown	(90)
let-7f	down	T2DM	Plasma of subjects with T2DM	Unknown	(90)
miRNA-92a-3p	up at mid gestation; down at late gestation	GDM	Plasma of subjects with GDM	Reduces the expression of NOS2 and SOCS5 and enhances insulin sensitivity in skeletal muscle	(47)
AC006064.4	up	GDM	Cord Blood subjects with GDM	Unknown	(91)
nc-HPS6-1:1	up	GDM	Cord Blood subjects with GDM	Unknown	(91)
nc-ZFHX3-7:1	up	GDM	Cord Blood subjects with GDM	Unknown	(91)

Reciprocally, the RNA cargo of EVs derived from immune cells is also functionally transferred to β-cells, confirming the existence of a two-way dialogue between pancreatic endocrine and immune cells. Actually, two miRNAs (miR-106b-5p and miR-222-3p) found in bone-marrow-derived EVs have been proposed as the main mechanism underlying the regeneration of β-cells caused by bone-marrow transplantation after pancreatic injury (92). On the other hand, a recent study reported the upregulation of a group of miRNAs (miR-142-3p, miR-142-5p and miR-155) in exosomes derived from T lymphocytes during the progression of insulitis in NOD mice (**Figure 2A**). Culture of mouse β-cells with T cell derived exosomes was sufficient to induce cell death, increasing Ccl2, Ccl7, and Cxcl10 expression through the regulation of NF-κB, while silencing of the aforementioned miRNAs protected β-cells from apoptosis (**Figure 2A**; **Table 1**) (77). These results demonstrate that miRNAs present in immune-derived EVs contribute to β-cell dismay and the pathogenesis of T1DM. Additional studies are warranted to fully characterize the role of miRNAs and other exosomal ncRNAs released by immune cells in β-cell survival and function.

### 3.2 EV associated ncRNAs mediated dialogue between immune cells and metabolic tissues

A crosstalk between β- and immune cells does not only occur in T1DM, but also in T2DM. Indeed, a hallmark of T2DM is low-grade chronic inflammation, in which resident and/or infiltrating macrophages play an important role in the development of the disease (93–95). A recent study showed that resident macrophages of pancreatic islets contribute to β-cell dysfunction during T2DM progression in an exosome-dependent manner. Qian and colleagues showed that exosomes derived from infiltrated M1-macrophages were enriched in miR-212-5p, which was transferred to β-cells (**Figure 2B**). Once incorporated, inhibited Sirtuin 2 and the downstream Akt/GSK-3β/β-catenin pathway, resulting in impaired insulin secretion (83). In order to investigate the mechanisms underlying macrophage accumulation in pancreatic islets, Sun et al. investigated β-cell derived exosomal miRNAs that were differentially expressed in pre-diabetic mice (characterized by hyperinsulinemia but normoglycemic) fed a high fat diet (HFD). One of the most upregulated miRNAs was miR-29a-3p which is highly expressed in metabolic tissues (75). Remarkably, miR-29b and miR-29a have also been implicated in the development of autoimmunity during T1DM (74, 76). β-cell specific miR-29a/b/c overexpressing transgenic mouse (βTG) showed higher levels of infiltrating macrophages, increased glucose intolerance and insulin resistance when compared to control mice but only under HFD or challenged with other metabolic disruptors such as streptozotocin (STZ) treatment and aging (**Table 1**). These results indicate that miR-29 is involved in stress-related processes. Exosomes derived from β-cells overexpressing miR-29 were enriched in this miRNA, which was then transferred to both infiltrated and circulating macrophages as well as monocytes. Once incorporated into macrophages, miR-29 upregulated MHC-II antigen presentation genes (H2-Aa, H2-Ab, and H2-Eb) and promoted polarization towards a pro-inflammatory M1-like phenotype (**Figure 2B**). The M1 polarization mediated by miR-29 through TRAF3, triggered not only islet infiltration but also macrophage migration to insulin-target tissues (liver, skeletal muscle and white adipose tissue; WAT), resulting in systemic inflammation and insulin resistance (75). Since miR-29b was involved in the process of autoimmunity in the NOD mice, it is tempting to speculate that this miRNA promotes the polarization to M1 also in the T1DM setting. Therefore, miR-29 could be an important player in the pathogenesis of both T1DM and T2DM.

Exosomal miRNAs released by macrophages infiltrated in insulin-target tissues also may affect their function and ability to clear blood glucose. Actually, exosomes derived from mouse macrophage RAW264.7 cells incubated with high glucose concentrations impaired glucose uptake and mitochondrial activity in 3T3-L1 adipocytes. This effect appeared to be mediated by miR-210 inhibition of NADH dehydrogenase ubiquinone 1 α subcomplex 4 (NDUFA4) (**Figure 2B**). Moreover, the authors showed that miR-210 knockout mice fed with a HFD gained less weight and showed enhanced glucose clearance compared to their wild-type (wt) counterparts (84). Likewise, exosomes derived from macrophages isolated from the bone marrow of HFD-fed mice and further polarized to M1, were shown to impair insulin action in hepatocytes *in vitro* as determined by reduced phosphorylation of AKT and GSK as well as glycogen synthesis. This process was mediated by miR-143-5p, which decreased the expression of the mitogen-activated protein kinase phosphatase-5 (Mkp5) (**Figure 2B**). In contrast, while exosomes derived from M1 macrophages may induce insulin resistance, exosomes derived from M2 macrophages have been shown to enhance insulin sensitivity in liver, skeletal muscle and WAT both *in vivo* and *in vitro*. These beneficial effects were mediated by exosomal miRNAs since exosomes derived from M2 macrophages from Dicer Knock-out mice (required for miRNA processing) did not show any effect on insulin signaling. More specifically, the authors found that miR-620 was the main driver of the improvement in insulin sensitivity. Actually, overexpressing this miRNA alone in miRNA-depleted exosomes was sufficient to recapitulate the effects on insulin signaling observed by the complete preparation of M2 macrophages derived-exosomes (**Figure 2B**) (85). Similarly, exosomes from other immune cells, such as natural killer cells (NK), have also been reported to contribute to insulin sensitivity. Wang and colleagues showed that the transfer of NK-derived exosomes from lean mice, decreased insulin resistance in mice fed with HFD *in vivo*. Mechanistically, miR-1249-3p, which was enriched in NK-derived exosomes from lean mice, was shown to be captured by AML12 (hepatocyte-derived cell line) and 3T3-L1 (adipocyte cell line) (**Figure 2B**). Once integrated into recipient cells, miR-1249-3p inhibits SKOR1, which results in reduced NF-κB activity, attenuating inflammation and insulin resistance (86).

Importantly, exosomal non-coding RNAs secreted by immune cells and then taken up by other immune cells, may exacerbate the inflammatory status associated with T2DM, worsening disease progression or its complications. Such an example is the lncRNA, MALAT1, which is increased in macrophage-derived exosomes incubated with high glucose (HG) concentrations. This exosomal lncRNA is functionally transferred to other macrophages where it acts as a sponge for miR-150-5p, subsequently increasing protein levels of resistin and reducing macrophage glucose uptake (**Figure 2B**) (62).

### 3.3 EV mediated crosstalk between organs involved in glucose homeostasis

**Figure 3:**
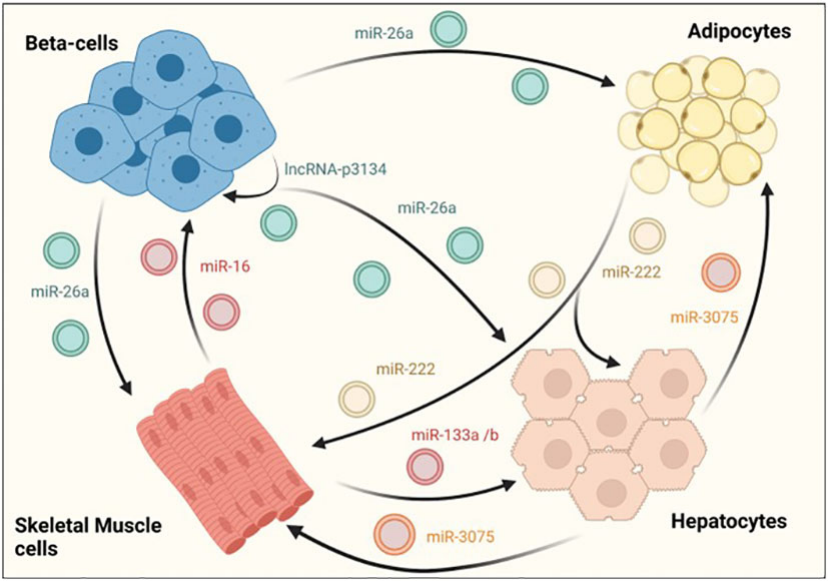
EV-mediated crosstalk between tissues regulating glucose homeostasis. β-cells secrete exosomes containing several RNA species, such as miR-26a that enhances insulin sensitivity in hepatocytes, white adipose tissue and skeletal muscle. On their part, exosomes derived from skeletal muscle cells also affect β–cell function and mass, demonstrating the existence of a two-way communication between pancreatic islets and insulin sensitive tissues. An intrincate crosstalk between hepatocytes, skeletal muscle and adipose tissue has also been demonstrated. For example, hepatocyte-derived exosomes containing miR-3075 affect the capacity of adipocytes and skeletal muscle cells to uptake glucose. Similarly, exosomes released from skeletal muscle cells may enhance hepatic insulin sensitivity through miR-133a/b. In contrast, miR-222 found in adipocyte-derived exosomes, impairs insulin action in other insulin target tissues.

Exosomes have also been shown to facilitate the crosstalk between different tissues involved in glucose homeostasis. In a recent study, Ji et al identified the hepatocyte exosomal miR-3075, that is involved in insulin resistance compensatory mechanisms in response to early weight gain leading to obesity. The authors showed that miR-3075 was highly enriched in hepatocyte-derived exosomes from mice fed with a HFD during 4 weeks but not after 16 weeks or lean animals. Exosomes enriched in this miRNA and injected in 12-week HFD animals accumulated in the liver, adipose tissue and skeletal muscle and restored insulin sensitivity, as assessed by AKT phosphorylation. Importantly, this increase in insulin signaling in metabolic tissues was translated into whole-body enhanced insulin sensitivity and glucose tolerance in treated mice (**Figure 3**) (96).

The RNA cargo of EVs derived from skeletal muscle have also contribute to compensatory mechanisms by regulating β-cell proliferation and subsequent increase in pancreatic islet area. Indeed, skeletal-muscle derived EVs from mice fed with a diet rich in palmitate (20%) (HPD-EVs) contained higher amounts of miR-16, which induced proliferation of MIN6B1 cells. Furthermore, miR-16 downregulated the expression of Ptch1, a receptor involved in pancreas development through the sonic hedgehog pathway (**Figure 3**) (87). Consistent with this, an increase in islet area along with altered expression of genes involved in development was also observed in mice fed with palmitate. In an independent study, skeletal-muscle derived exosomes were found to mediate beneficiary effects produced by physical exercise through miR-133a and miR-133b (**Figure 3**). These miRNAs were shown to be incorporated by hepatocytes, increasing the expression of forkhead box O1 (FoxO1) and enhancing hepatic insulin sensitivity (97).

Interestingly, it has been reported that adipose tissue is one of the major sources of total circulating exosomes in the body (98). However, there are few studies analyzing the effect of non-coding RNAs associated in adipocyte-derived exosomes. Li and colleagues recently reported an increase in mir-222 levels in WAT from obese patients with insulin resistance and in serum samples from individuals with T2DM. Furthermore, serum exosomes and gonadal WAT of HFD-fed mice also showed increased levels of mir-222 that correlated with glucose intolerance and insulin resistance as determined by decreased levels of phospho-AKT in liver and skeletal muscle tissues (**Figure 3**) (88).

Further supporting the existence of an exquisitely regulated dialogue between different metabolic tissues, β-cell derived miRNAs were shown to enhance insulin sensitivity in a paracrine manner. Recently, it was demonstrated that exosomal miR-26a secreted by β-cells alleviates insulin resistance and hyperinsulinemia in HFD-fed mice (**Figure 3**; **Table 1**). Furthermore, miR-26a was reduced in serum exosomes of overweight humans and was inversely correlated with HOMA-IR as well as fasting glucose and insulin levels (99). Another exosomal ncRNA that could potentially participate in the crosstalk between β-cells and insulin target tissues is lncRNA-p3134. This lncRNA is secreted from MIN6 and mouse islets when cultured with high glucose concentrations, consistent with serum exosomes from diabetic patients having a 4-fold enrichment when compared to healthy subjects (**Table 1**). Upregulating lncRNA-p3134 levels by intravenous hydrodynamic injections in db/db mice enhanced glucose clearance and insulin secretion in response to a glucose load, while *in vitro* studies showed that exosomes containing this lncRNA stimulated GSIS in MIN6 cells (**Figure 3**). Beneficial effects of lncRNA-p3134 were mediated through the activation of the insulin PI3K/Akt/mTOR signaling pathway. Therefore, although this study focused on β-cells, it is tempting to speculate that the exosomal lncRNA-p3134 may also activate the insulin signaling PI3K/Akt pathway in target tissues (89).

### 3.4 EV associated ncRNAs as biomarkers in Diabetes Mellitus

The presence of EVs as a secretory product in many biological fluids such as blood, milk, urine and saliva, prompted numerous studies assessing their possible role as novel biomarkers. In the last decade, circulating EVs number, size and content has been found to be altered in several diseases. Therefore, EVs are not only important mediators in cell-to-cell communication but also represent a possible source of biomarkers (100, 101). More specifically, EV-associated non-coding RNAs could be ideal biomarkers as their expression is tightly regulated in a cell-type/time specific manner (102).

#### 3.4.1 Type 1 Diabetes Mellitus

Currently, there are more than 1.6 million children and adolescents living with T1DM and the prevalence of this diseases is increasing (103). Normally, clinical symptoms of T1DM are preceded by a long latency period, where patients are asymptomatic but still present β-cell dysfunction and death as well as autoantibodies against islet autoantigens. Actually, T1DM is diagnosed when there is a 60-80% reduction in the functional β-cell mass (104). Currently, susceptibility genes, such as high-risk HLA genes and islet autoantibodies (GAD65, IA-2, pro-insulin, zinc transporter-8 (ZnT8), islet-specific glucose-6-phosphatase catalytic subunit related protein (IGRP), imogen-38, pancreatic duodenal homeobox factor 1 (PDX1), chromogranin A (CHGA) and islet amyloid polypeptide (IAPP)) are the gold-standard approaches for the detection of T1DM. However, a clinical study recently reported that less than 50% of cases are identified by genetic marker screening, while a major limitation of diagnostic autoantibodies is that they appear relatively late during disease progression (46).

In an attempt to identify novel biomarkers for T1DM, Garcia-Contreras et al. assessed the expression of plasma-derived exosome miRNAs in long-term T1DM (25.3 ± 15.9 years) and healthy individuals. The authors identified 7 different miRNAs that were differentially expressed in T1DM individuals. Six of these miRNAs (miR-16, miR-302d-3p, miR-378e, miR-570–3p, miR-574-5p, miR-579) were down-regulated, while miR-25-3p was upregulated in T1DM subjects. However, only miR-16-5p, miRNA-302d-3p, miR-574-5p showed differential expression when analyzed by qRT-PCR (**Table 1**) (40). Interestingly, miR-16-5p was recently found to inhibit β-cell apoptosis by targeting CXCL10, while miR-574-5p has also been proposed as a biomarker for GDM (78–80). An independent study, identified three additional exosomal miRNAs (miR-193b-5p, miR-122-5p, and miR-445-5p) that could serve as biomarkers for different stages of T1DM since they were differentially expressed between recently diagnosed and long-term (10 years) T1DM individuals. Furthermore, the authors also showed that miR-195-3p, miR-455-5p and miR-185-5p were differentially expressed between healthy individuals and a cohort comprised of individuals with at least 10-year since diagnosis of T1DM (76). Lakhter and colleagues also proposed the exosomal miRNA, miR-21-5p, as a novel biomarker for T1DM since it was secreted in MIN6 derived EVs and the human β-cell line EndoC-βH1 in response to cytokine treatment. In addition, the expression of this miRNA was progressively increased in serum exosomes from NOD mice 3 weeks prior the onset of diabetes. More importantly, these results were validated in humans, since serum-derived exosomal miR-21-5p was increased by 3-fold in recently diagnosed children with T1DM as compared to healthy counterparts (**Table 1**) (81). Further studies should be performed to confirm whether these miRNAs are reliable biomarkers and to determine whether gender, age, weight or other factors may also influence their expression. These factors, together with the relatively small size of the cohorts, could contribute to inconsistent results between studies which will explain the differences in the miRNAs identified by different studies.

Recently, Pang et al., analysed the profile of exosomal lncRNAs in 10 individuals with T1DM and 10 healthy subjects. The authors found 162 lncRNAs that were differentially expressed between the two experimental groups in the RNA-seq data. However, differential expression of selected lncRNAs was not validated by qRT-PCR (**Table 1**) (82). Therefore, more studies are needed to identify exosomal lncRNAs that can truly be used as biomarkers for T1D.

#### 3.4.2 Type 2 Diabetes Mellitus

The prevalence and incidence of T2DM, representing >90% of all cases of DM, is reaching epidemic proportions and the number of people with DM is expected to rise from 537 million adults in 2021 to 783 million by 2045 (103). Therefore, identification of individuals at high risk of developing T2DM using alternative prognostic tools or biomarkers such ncRNA is of great importance as early interventions may refrain disease development. Towards this goal, several studies have identified different miRNAs and other ncRNAs that are differentially expressed in subjects with T2DM and healthy controls.

Santovito et al. reported 25 miRNAs differentially expressed in exosomes from T2DM individuals: four upregulated and 21 downregulated. Among these miRNAs, the authors confirmed by qRT-PCR the expression of miR-326, let-7a and let-7f which had predicted targets involved in the development of T2DM (90).

Interestingly, MALAT1 was found to be significantly reduced in serum-derived EVs from individuals with metabolic syndrome (MetS), that agglomerates the co-occurrence of a number of conditions such as central obesity, dislipedimia and hypertension that greatly increase the risk of T2DM and cardiovascular diseases (105). Furthermore, a tendency towards decreased MALAT1 levels was also observed in serum-derived EVs from T2DM individuals when compared to controls (**Table 1**) (106). These results suggest that MALAT1 could be a biomarker for metabolic syndrome, although further studies analyzing the expression of this lncRNA during different stages of metabolic syndrome, prediabetes and T2D should be performed.

Other studies have focused on identifying biomarkers that could predict the future development of T2DM in animal models of obesity, which is a major risk factor for this disease. In this line, Castaño et al., identified several miRNAs (miR-122, miR-192, miR-27a-3p, and miR-27b-3p) that were differentially expressed in the blood of obese HFD-fed mice compared to lean control animals. Remarkably, administration of exosomes from HFD-fed mice to lean animals, induced glucose intolerance, while exosomes from lean mice containing mimetics of the 4 miRNAs identified had a similar effect in glucose homeostasis (107). In a more recent study, the profile of exosomal circulating miRNAs was determined in healthy volunteers (HV), individuals with obesity without T2DM and obese people with T2DM. The authors found that there were 25 miRNAs (upregulated=14 and downregulated=11) differentially expressed between obese subjects with T2DM and without T2DM. Intriguingly, mir-let7-b which was upregulated in a T1DM model, was found to be downregulated in T2DM individuals compared with obese non-diabetic subjects. Furthermore, other members of mir-let7 family, were also differentially expressed between HV and obese individuals. Interestingly, miR-26a was also identified as a possible biomarker since it was upregulated in obese subjects without T2DM compared with T2DM individuals (108). These results were consistent with the study by Xu et al. that showed that miR-26a expression was reduced in plasma derived exosomes from T2DM individuals and that it promoted insulin sensitivity (99). Therefore, combining both studies, it is tempting to speculate that miR-26a may serve as a biomarker for T2DM but also a good candidate to explore as a therapeutic target. Actually, increased expression of this miRNA in obese people without T2DM might serve as a compensatory mechanism, helping to maintain glucose tolerance and insulin sensitivity. Intriguingly, an independent study found miR-26a increased in the serum of children with T1DM (109).

#### 3.4.3 Gestational Diabetes Mellitus

It is estimated that one in six pregnancies (21 million) are affected by DM (103). However, no many studies have addressed the role of EV associated non-coding RNAs in GDM and only a few reports have characterized the profile of exosomal miRNAs and lncRNA species in an attempt to find biomarkers that could predict the development of the disease. In this line, Nair and colleagues found 101 miRNAs that showed altered expression between healthy control and GDM subjects. Among these miRNAs, they identified miRNA-92a-3p as a specific factor enhancing insulin sensitivity in skeletal muscle biopsies from pregnant women *in vitro* (**Table 1**) (47). Another microRNA that enhances insulin sensitivity, miR-574-5p (previously proposed as a T1DM biomarker), was also recently found to be reduced in serum from women with GDM (**Table 1**) (78, 79). Another study that was published recently found 98 mRNAs, 372 lncRNAs, and 452 circRNAs differentially expressed in cord blood exosomes from females with GDM compared to healthy controls. However, only the differential expression of three lncRNAs (AC006064.4, nc-HPS6-1:1, nc-ZFHX3-7:1) and one circRNA (circ_0014635) were validated by qRT-PCR. Furthermore, only AC006064.4 served as a good prediction biomarker for macrosomia, which is one of the most common complications in GDM (**Table 1**) (91).

## 4 Conclusions

Non-coding RNAs associated with EVs are clearly involved in multiple mechanisms, mediating the communication between pancreatic and immune cells as well as insulin target tissues in both T1DM and T2DM. Alterations in such EV-mediated communication have been shown to play an important role in the autoimmune process during T1DM as well as in the development of insulin resistance and β-cell dysfunction during T2DM. Several miRNA families, such as miR-29 and miR-let7, have been associated with both T1DM and T2DM (70, 90, 94), suggesting that both diseases may share common etiological features. Indeed, for example the role of miR-29 in macrophage M1 polarization in T2DM could likely be extrapolated to T1DM, while further studies need to determine the role of miR-let7 in T1DM and T2DM. Furthermore, whether these miRNAs are also involved in GDM needs to be further studied.

Several ncRNAs identified to be differentially expressed during T2DM are part of compensatory mechanisms that take place in situations of metabolic stress, improving β-cell function and/or enhancing insulin sensitivity (97, 106). Therefore, ncRNAS found in EVs could be of value as novel therapeutic targets to treat T2DM. Particularly interesting in this regard, may be the study of miR-26a which is increased in healthy obese subjects but decreased in T2DM individuals and which has been shown to modulate insulin sensitivity (99).

EVs associated ncRNAs have also been proposed as a potential source of disease biomarkers. Indeed, studies characterizing the small or long RNA profile of serum and plasma derived exosomes have found several miRNAs and lncRNAs that could serve as biomarkers for T1DM, T2DM and GDM.

However, there are still multiple critical issues surrounding EV-associated ncRNAs in DM research. Indeed, there is still a need to define a rigorous criterion for quality control and standardized strategies for quantification and characterization of EVs. Furthermore, most studies have focused on exosomal miRNAs, while the role of other ncRNA species as mediators in the communication of immune cells and metabolic tissues is largely unknown. In conclusion, EVs represent a relatively novel and still largely unexplored research field, which could be exploited to find novel players in disease progression, diagnostic and prognosis biomarkers as well as therapeutic agents.

## Author contributions

LL-N and BG jointly conceived and wrote the manuscript. LL-N provided the original draft of the article and prepared the figures. NC-V contributed to the structure, literature research and writing of the manuscript. All authors contributed to the article and approved the submitted version.
